# Complete Genome Sequence and Annotation of the Paracoccus pantotrophus Type Strain DSM 2944

**DOI:** 10.1128/MRA.01290-19

**Published:** 2020-01-02

**Authors:** Julia A. Bockwoldt, Martin Zimmermann, Till Tiso, Lars M. Blank

**Affiliations:** a Institute of Applied Microbiology, RWTH Aachen University, Aachen, Germany; b Chair of Technical Microbiology, Technical University of Munich, Freising, Germany; University of Southern California

## Abstract

Paracoccus spp. are metabolically versatile alphaproteobacteria able to perform heterotrophic and chemoautotrophic growth. This study describes the whole-genome sequence of the Paracoccus pantotrophus type strain DSM 2944 (ATCC 35512, LMD 82.5, GB17). The genome sequence revealed the presence of a complete *phaZ phaC phaP phaR* gene cluster related to polyhydroxyalkanoate metabolism.

## ANNOUNCEMENT

Paracoccus pantotrophus DSM 2944^T^ (formerly designated Thiosphaera pantotropha) was isolated in 1983 from a denitrifying, sulfide-oxidizing effluent treatment plant in Delft, Netherlands. The strain is a nonmotile, facultative anaerobic, nonpathogenic, Gram-negative coccus occurring singly, pairwise, or as short chains in spherical to rod-shaped forms (1–3). *P. pantotrophus* DSM 2944^T^ belongs to the family *Rhodobacteraceae* in the phylum *Alphaproteobacteria*. This strain can grow chemoautotrophically with carbon dioxide and hydrogen or reduced sulfur compounds and is capable of denitrification. Under nitrogen limitation, polyhydroxybutyrate can be produced (1, 4, 5). The versatile metabolic pathways render *P. pantotrophus* DSM 2944^T^ a promising strain for biotechnological applications.

Strain DSM 2944^T^ was obtained from the German Collection of Microorganisms and Cell Cultures (DSMZ, Braunschweig, Germany) and was grown aerobically in lysogeny broth (LB) medium at 30°C to early exponential phase (6). Genomic DNA was extracted with the standard phenol-chloroform method, followed by construction of a 16-kb library. The sequencing of the whole genome of *P. pantotrophus* DSM 2944^T^ was performed using the PacBio RS II platform (Menlo Park, CA, USA) with a 240-min movie, obtaining 70,835 total reads, an average read length of 10,089 bp, an average reference coverage of 147-fold, and an *N*_50_ value of 14,250 bp. The size of the whole genome was found to be 4,409,379 bp, with a G+C content of 67.7%. The raw genome sequence data were proofed and assembled with SMRT v6.0.0 by using one single-molecule real-time (SMRT) cell featuring HGAP v3 (7), resulting in four circularized DNA elements comprising 2,248,278 bp, 1,525,820 bp, 535,332 bp, and 99,949 bp with coverages of 149-fold, 139-fold, 168-fold, and 157-fold, respectively. Circularization was confirmed by fusing overlapping sequences outside open reading frame regions. Pulsed-field gel electrophoresis was performed following the protocol of Winterstein and Ludwig and verified the presence of four DNA fragments in DSM 2944^T^, which corresponded with Winterstein and Ludwig’s findings of four DNA fragments of similar sizes (8). Furthermore, the two smaller DNA fragments were already described as megaplasmids named pPAN2 (pHG41), with a size of 535,332 bp, and pPAN1 (pHG42), with a size of 99,949 bp. For the plasmid pPAN1, a partial sequence was recovered with a size of 2,005 bp (2%), and for pPAN2, no sequence information was available (9–12). The two larger DNA elements are assumed to be chromosomes, as the presence of up to three chromosomes within the genus of *Paracoccus* is already known (8). Furthermore, essential and housekeeping genes, for example, 16S rRNA genes, genes for heat shock proteins (*dnaK* and *dnaJ*), genes involved in chromosomal replication (*dnaA* and *dnaE*), and cell division genes (*ftsZ* and *ftsA*), were identified only within the assumed chromosomal sequences (13, 14). The genome annotation was performed with the NCBI Prokaryotic Genome Annotation Pipeline (PGAP) (15, 16). The genome of *P. pantotrophus* DSM 2944^T^ contains a total of 4,076 coding sequences, including three 16S rRNA, three 23S rRNA, and 51 tRNA genes. The *phaZ phaC phaP phaR* gene cluster associated with polyhydroxyalkanoate (PHA) metabolism was identified on the larger putative chromosome, including PHA depolymerase (*phaZ*), PHA synthase class I (*phaC*), PHA granule-associated phasin (*phaP*), and PHA synthesis repressor (*phaR*), and was previously described for Paracoccus denitrificans (17), which has also been demonstrated to produce poly-β-hydroxybutyrate (PHB) from alcohols under nitrogen limitation (18, 19) and is closely related to *P. pantotrophus*. Furthermore, the formation of PHB and other PHA species, for example, polyhydroxyvalerate, by *P. pantotrophus* was described (5). The sequences described here confirm the capability of *P. pantotrophus* DSM 2944^T^ to produce PHA under certain conditions.

### Data availability.

The genome sequence of *P. pantotrophus* DSM 2944^T^ has been deposited in GenBank under the accession numbers CP044423 (smaller chromosome), CP044424 (pPAN1 [pHG42]), CP044425 (pPAN2 [pHG41]), and CP044426 (larger chromosome). PacBio sequencing reads for *P. pantotrophus* DSM 2944^T^ are deposited in the SRA and available under accession number SRR10314275.
